# Association of Hospital-Level Acute Resuscitation and Postresuscitation Survival With Overall Risk-Standardized Survival to Discharge for In-Hospital Cardiac Arrest

**DOI:** 10.1001/jamanetworkopen.2020.10403

**Published:** 2020-07-10

**Authors:** Saket Girotra, Brahmajee K. Nallamothu, Yuanyuan Tang, Paul S. Chan

**Affiliations:** 1 Division of Cardiovascular Diseases, Department of Internal Medicine, University of Iowa Carver College of Medicine, Iowa City; 2 Center for Access and Delivery Research and Evaluation, Iowa City Veterans Affairs Medical Center, Iowa City, Iowa; 3 Center for Clinical Management Research, Ann Arbor Veterans Affairs Medical Center, Ann Arbor, Michigan; 4 Department of Internal Medicine, University of Michigan Medical School, Ann Arbor; 5 Saint Luke’s Mid America Heart Institute and the University of Missouri, Kansas City

## Abstract

**Importance:**

Survival after in-hospital cardiac arrest depends on 2 distinct phases: responsiveness and quality of the hospital code team (ie, acute resuscitation phase) and intensive and specialty care expertise (ie, postresuscitation phase). Understanding the association of these 2 phases with overall survival has implications for design of in-hospital cardiac arrest quality measures.

**Objective:**

To determine whether hospital-level rates of acute resuscitation survival and postresuscitation survival are associated with overall risk-standardized survival to discharge for in-hospital cardiac arrest.

**Design, Settings, and Participants:**

This observational cohort study included 86 426 patients with in-hospital cardiac arrest from January 1, 2015, through December 31, 2018, recruited from 290 hospitals participating in the Get With The Guidelines–Resuscitation registry.

**Exposures:**

Risk-adjusted rates of acute resuscitation survival, defined as return of spontaneous circulation for at least 20 minutes, and postresuscitation survival, defined as survival to discharge among patients achieving return of spontaneous circulation.

**Main Outcomes and Measures:**

The primary outcome was overall risk-standardized survival rate (RSSR) for in-hospital cardiac arrest calculated using a previously validated model. The correlation between a hospital’s overall RSSR and risk-adjusted rates of acute resuscitation and postresuscitation survival were examined.

**Results:**

Of 86 426 patients with in-hospital cardiac arrest, the median age was 67.0 years (interquartile range [IQR], 56.0-76.0 years); 50 665 (58.6%) were men, and 71 811 (83.1%) had an initial nonshockable cardiac arrest rhythm. The median RSSR was 25.1% (IQR, 21.9%-27.7%). The median risk-adjusted acute resuscitation survival was 72.4% (IQR, 67.9%-76.9%), and risk-adjusted postresuscitation survival was 34.0% (IQR, 31.5%-37.7%). Although a hospital’s RSSR was correlated with survival during both phases, the correlation with postresuscitation survival (ρ, 0.90; *P* < .001) was stronger compared with the correlation with acute resuscitation survival (ρ, 0.50; *P* < .001). Of note, there was no correlation between risk-adjusted acute resuscitation survival and postresuscitation survival (ρ, 0.09; *P* = .11). Compared with hospitals in the lowest RSSR quartile, hospitals in the highest RSSR quartile had higher rates of acute resuscitation survival (75.4% in quartile 4 vs 66.8% in quartile 1; *P* < .001) and postresuscitation survival (40.3% in quartile 4 vs 28.7% in quartile 1; *P* < .001), but the magnitude of difference was larger with postresuscitation survival.

**Conclusions and Relevance:**

The findings suggest that hospitals that excel in overall in-hospital cardiac arrest survival, in general, excel in either acute resuscitation or postresuscitation care but not both; efforts to strengthen postresuscitation care may offer additional opportunities to improve in-hospital cardiac arrest survival.

## Key Points

**Question:**

Are rates of acute resuscitation and postresuscitation survival associated with rates of overall risk-standardized survival to discharge for in-hospital cardiac arrest?

**Findings:**

In this cohort study of 86 426 patients with in-hospital cardiac arrest from 290 hospitals, a hospital’s overall risk-standardized survival rate was more strongly correlated with its risk-adjusted postresuscitation survival than with acute resuscitation survival. There was no correlation between risk-adjusted acute resuscitation and postresuscitation survival.

**Meaning:**

The findings suggest that, because current quality improvement initiatives focus largely on acute resuscitation care, efforts to strengthen postresuscitation care may offer additional opportunities to improve survival after in-hospital cardiac arrest.

## Teaser

This cohort study evaluates patients’ overall risk-standardized survival rate for in-hospital cardiac arrest and examines its association with risk-adjusted rates of acute resuscitation and postresuscitation survival.

## Introduction

There is substantial variation between hospitals for survival of in-hospital cardiac arrest (IHCA).^1,2^ To date, most quality improvement initiatives have focused on delivering timely chest compressions, early defibrillation, and epinephrine during an acute resuscitation response.^3,4,5,6,7^ However, what has been underappreciated is that IHCA survival depends on 2 distinct phases of care.^8,9^ Survival may depend on care during the initial resuscitation, which is largely associated with the responsiveness and quality of the hospital resuscitation or code team (ie, acute resuscitation phase). Survival may also depend on care after return of spontaneous circulation, driven largely by the quality and expertise of intensive and specialty care at a hospital (ie, postresuscitation phase).

Previous studies^1,8^ of IHCA have not defined the association of acute resuscitation and postresuscitation phases with overall survival. Although studies have shown that overall survival for IHCA varies by more than 3-fold across hospitals,^1,2^ it remains unknown whether high survival at top-performing hospitals is associated with high rates of acute resuscitation survival, postresuscitation survival, or both. This is important to understand because current initiatives for improving resuscitation care quality and reducing variation in IHCA survival largely focus on incentivizing acute resuscitation care delivery, such as reducing time to defibrillation and delivering effective chest compressions. However, such initiatives will have the strongest association with survival if hospitals that excel in acute resuscitation care also excel in postresuscitation care.

To address this gap in knowledge, we used contemporary data from the American Heart Association Get With The Guidelines (GWTG)–Resuscitation registry^10^ to examine site-level variation in IHCA survival to identify hospitals that had high overall survival rates among patients with IHCA after adjustment for patient case mix. We further examined the extent of correlation between a hospital’s overall IHCA survival with its risk-adjusted rate of acute resuscitation survival and postresuscitation survival. We believe that a better understanding of the association of overall IHCA survival with acute resuscitation and postresuscitation survival will have important implications for designing future initiatives for improving resuscitation care quality.

## Methods

### Study Design and Data Sources

We designed a cohort study within the GWTG-Resuscitation registry, a prospective multisite registry of IHCA events in the US. The design of this registry has been described previously.^10^ The study was reviewed by the University of Iowa institutional review board, Iowa City, which waived the requirement for informed consent because of the use of deidentified data and approved the study. This study followed the Strengthening the Reporting of Observational Studies in Epidemiology (STROBE) reporting guideline.

The design of the GWTG-Resuscitation registry has been described previously.^10^ In brief, all hospitalized patients with confirmed IHCA, defined as absence of a palpable central arterial pulse, apnea, and unresponsiveness, and without do-not-resuscitate orders, were enrolled by trained personnel at participating hospitals. Multiple case-finding approaches were used, including review of centralized collection of cardiac arrest flow sheets, routine review of code cards, pharmacy tracer drug records, review of hospital paging system logs, and hospital billing charges for resuscitation medications. Hospital participation was voluntary with data collected using standardized Utstein-style definitions for all patient variables and outcomes to facilitate uniform reporting across hospitals.^11,12^ Accuracy and completeness of the data were ensured by rigorous training and certification of medical staff at participating hospitals along with use of standardized software for internal checks and periodic reabstractions and audits of collected data.^10^

### Study Population

Using contemporary data from GWTG-Resuscitation, we identified 90 343 patients aged 18 years or older who experienced an index IHCA event from January 1, 2015, through December 31, 2018. From this sample, we excluded patients who were missing data on comorbidities (n = 546), arrest location (n = 53), and survival (n = 1042). To ensure that the estimates of hospital survival for IHCA obtained from multivariable models were statistically reliable, we excluded 2276 patients from hospitals with fewer than 50 cases during the study period. Our final cohort comprised 86 426 patients at 290 hospitals (eFigure 1 in the Supplement).

### Study Variables and Outcomes

The main outcomes of our study were a hospital’s overall risk-standardized survival rate (RSSR) to discharge and its 2 components: acute resuscitation survival and postresuscitation survival. Acute resuscitation survival was defined as return of spontaneous circulation for at least 20 minutes among patients with an initial cardiac arrest. Postresuscitation survival was defined as survival to discharge among patients who achieved return of spontaneous circulation.

Patient level data included (1) demographics (age, sex, and race/ethnicity); (2) comorbidities and preexisting medical conditions (current or previous heart failure; current or previous myocardial infarction; diabetes; renal, hepatic, or respiratory insufficiency; baseline evidence of motor, cognitive, or functional deficits [central nervous system depression]; acute stroke; pneumonia; hypotension; sepsis; major trauma; metabolic or electrolyte abnormalities; or metastatic or hematologic malignant neoplasm); (3) cardiac arrest characteristics (initial rhythm [asystole, pulseless electrical activity, ventricular fibrillation, and pulseless ventricular tachycardia]); (4) the use of a hospital-wide cardiopulmonary arrest alert; (5) time of cardiac arrest (weekday: 8:00 am to 5:00 pm from Monday to Friday, weeknight: 5:00 pm to 8:00 am from Monday to Friday, and weekend: all day on Saturday and Sunday); (6) cardiac arrest location (intensive care unit [ICU], monitored unit, nonmonitored unit, emergency department, procedural or surgical area, and other); and (7) interventions in place at the time of cardiac arrest (mechanical ventilation, use of intravenous vasoactive vasopressors, intraarterial lines, and dialysis). Hospital-level variables included number of beds, number of ICU and cardiac ICU beds, academic status, urban or rural location, and geographic census region.

### Statistical Analyses

Our primary objective was to assess the extent of correlation between a hospital’s overall rate of survival to discharge for IHCA with its acute resuscitation and postresuscitation survival. For the outcome of hospital rate of survival to discharge, we calculated the overall RSSR for each hospital in the cohort by using a previously validated method.^1^ Specifically, we built a 2-level multivariable hierarchical regression model to relate the log odds of survival with patient variables. Hierarchical models account for clustering of patients within a hospital and separate within-hospital variation from between-hospital variation as well as model the assumption that underlying differences in hospital quality explain the between-hospital differences in survival.^13^ Patient variables included in this model were based on the previous validation study and included age, initial cardiac arrest rhythm, location of arrest, hypotension, sepsis, metastatic or hematologic malignanc neoplasm, hepatic insufficiency, mechanical ventilation, and use of intravenous vasopressors before the cardiac arrest. The hospital site was included as a random effect in these models.

Using regression coefficients from this model, we estimated each hospital’s risk-standardized survival as the ratio of predicted to expected survival multiplied by the overall unadjusted survival rate for patients with IHCA. Compared with the observed to expected ratio, the predicted to expected ratio does not unfairly penalize small-volume hospitals by accounting for the lower precision in survival estimates from such volume hospitals.^14^ This model had excellent discrimination (C statistic, 0.74) and calibration in the previous validation study.^1^ We also quantified variation in survival rates across hospitals using median odds ratios from the the hierarchical models described using the variance estimate of the random hospital intercept.^15^

Because validated models to risk standardize acute resuscitation and postresuscitation survival have not been developed, we calculated each hospital’s risk-adjusted rate of acute resuscitation survival and postresuscitation survival. Risk adjustment was performed using the same variables that were included in the model for overall survival.

Next, we categorized study hospitals into quartiles based on their overall RSSR (Q1, lowest quartile; Q4, highest quartile) and used descriptive statistics to compare hospital-level and patient characteristics using a χ^2^ test for categorical variables and analysis of variance for linear variables. We then compared rates of acute resuscitation survival and postresuscitation survival across hospital quartiles of RSSR and calculated the absolute difference between Q4 and Q1 RSSR quartiles. We also examined the extent to which hospital performance on the RSSR metric was concordant with performance on the acute resuscitation and postresuscitation survival. Finally, we calculated the Pearson correlation between hospital’s RSSR and its risk-adjusted rates of acute resuscitation survival and postresuscitation survival. The significance level was set at *P* < .05 using a 2-sided test. All analyses were conducted using SAS, version 9.4 (SAS Institute Inc).

## Results

A total of 290 hospitals and 86 426 patients with IHCA were included. Table 1 shows baseline characteristics of patients with IHCA in our study. Overall, the median age was 67.0 years (interquartile range, 56.0-76.0 years); 50 665 (58.6%) were men, and 58 708 (67.9%) were white. An initial nonshockable cardiac arrest rhythm of asystole or pulseless electrical activity was present in 71 811 patients (83.1%). Nearly half (41 937 [48.5%]) of the arrests occurred in an ICU, and 36 134 patients (41.8%) were receiving mechanical ventilation at the time of cardiac arrest. Table 2 shows the baseline characteristics of study hospitals. The median IHCA case volume was 234.0 (IQR, 109.0-393.0) cardiopulmonary arrest events. Study hospitals were evenly distributed according to census regions and bed size. Most of the hospitals (217 of 233 [93.1%]) were located in an urban area, and 141 of 233 (60.5%) were teaching hospitals.

**Table 1.** Patient Characteristics^a^

Characteristic	Total (N = 86 426)	Overall risk-standardized survival rate				*P* value^b^
		Quartile 1 (n = 19 423)	Quartile 2 (n = 21 859)	Quartile 3 (n = 21 987)	Quartile 4 (n = 23 157)	
Year of admission						<.001
2015	19 272 (22.3)	4716 (24.3)	4738 (21.7)	4528 (20.6)	5290 (22.8)	
2016	21 755 (25.2)	4819 (24.8)	5344 (24.4)	5628 (25.6)	5964 (25.8)	
2017	23 364 (27.0)	5194 (26.7)	5696 (26.1)	6102 (27.8)	6372 (27.5)	
2018	22 035 (25.5)	4694 (24.2)	6081 (27.8)	5729 (26.1)	5531 (23.9)	
Age at admission, y						<.001
Mean (SD)	65.2 (15.5)	65.6 (15.5)	64.9 (15.5)	65.0 (15.5)	65.4 (15.3)	
Median (IQR)	67.0 (56.0-76.0)	67.0 (56.0-77.0)	66.0 (56.0-76.0)	66.0 (56.0- 76.0)	67.0 (57.0-76.0)	
Sex						<.001
Male	50 665 (58.6)	11 160 (57.5)	12 919 (59.1)	12 864 (58.5)	13 722 (59.3)	
Female	35 761 (41.4)	8263 (42.5)	8940 (40.9)	9123 (41.5)	9435 (40.7)	
Race/ethnicity						<.001
White	58 708 (67.9)	10 902 (56.1)	15 715 (71.9)	14 963 (68.1)	17 128 (74.0)	
Black	19 734 (22.8)	6067 (31.2)	4311 (19.7)	5019 (22.8)	4337 (18.7)	
Other	2271 (2.6)	577 (3.0)	553 (2.5)	586 (2.7)	555 (2.4)	
Unknown	5713 (6.6)	1877 (9.7)	1280 (5.9)	1419 (6.5)	1137 (4.9)	
Initial rhythm during cardiac arrest						
Asystole	22 062 (25.5)	5808 (29.9)	5692 (26.0)	5173 (23.5)	5389 (23.3)	<.001
Pulseless electrical activity	49 749 (57.6)	10 857 (55.9)	12 322 (56.4)	13 089 (59.5)	13 481 (58.2)	
Ventricular fibrillation	8137 (9.4)	1628 (8.4)	2059 (9.4)	2142 (9.7)	2308 (10.0)	
Pulseless ventricular tachycardia	6478 (7.5)	1130 (5.8)	1786 (8.2)	1583 (7.2)	1979 (8.5)	
Unit location						<.001
Intensive care	41 937 (48.5)	9812 (50.5)	10 683 (48.9)	10 251 (46.6)	11 191 (48.3)	
Monitored	12 655 (14.6)	2826 (14.5)	3403 (15.6)	2690 (12.2)	3736 (16.1)	
Nonmonitored	12 820 (14.8)	2851 (14.7)	2856 (13.1)	4072 (18.5)	3041 (13.1)	
Emergency department	10 776 (12.5)	2450 (12.6)	2915 (13.3)	2836 (12.9)	2575 (11.1)	
Procedural	6701 (7.8)	1114 (5.7)	1659 (7.6)	1749 (8.0)	2179 (9.4)	
Other	1537 (1.8)	370 (1.9)	343 (1.6)	389 (1.8)	435 (1.9)	
Time of cardiac arrest						.06
Weekday	43 859 (51.1)	9704 (50.3)	11 081 (51.0)	11 192 (51.3)	11 882 (51.7)	
Weeknight	15 300 (17.8)	3555 (18.4)	3816 (17.6)	3891 (17.8)	4038 (17.6)	
Weekend	26 652 (31.1)	6047 (31.3)	6811 (31.4)	6737 (30.9)	7057 (30.7)	
Missing	615	117	151	167	180	
Preexisting condition						
Current heart failure	12 748 (14.8)	2438 (12.6)	3074 (14.1)	3145 (14.3)	4091 (17.7)	<.001
Prior heart failure	19 757 (22.9)	4062 (20.9)	4659 (21.3)	4950 (22.5)	6086 (26.3)	<.001
Current myocardial infarction	12 573 (14.5)	2515 (12.9)	2946 (13.5)	3251 (14.8)	3861 (16.7)	<.001
Prior myocardial infarction	11 862 (13.7)	2288 (11.8)	2577 (11.8)	3064 (13.9)	3933 (17.0)	<.001
Diabetes	30 034 (34.8)	6638 (34.2)	7546 (34.5)	7537 (34.3)	8313 (35.9)	<.001
Renal insufficiency	31 694 (36.7)	7128 (36.7)	7752 (35.5)	7958 (36.2)	8856 (38.2)	<.001
Hepatic insufficiency	7591 (8.8)	1345 (6.9)	1888 (8.6)	1970 (9.0)	2388 (10.3)	<.001
Respiratory insufficiency	41 341 (47.8)	8264 (42.5)	9785 (44.8)	10 786 (49.1)	12 506 (54.0)	<.001
Baseline CNS depression	6380 (7.4)	1313 (6.8)	1393 (6.4)	1689 (7.7)	1985 (8.6)	<.001
Acute stroke	3499 (4.0)	783 (4.0)	907 (4.1)	776 (3.5)	1033 (4.5)	<.001
Acute nonstroke CNS event	7167 (8.3)	846 (4.4)	1934 (8.8)	2025 (9.2)	2362 (10.2)	<.001
Pneumonia	12 330 (14.3)	2614 (13.5)	2956 (13.5)	3325 (15.1)	3435 (14.8)	<.001
Hypotension	23 913 (27.7)	3509 (18.1)	5584 (25.5)	6634 (30.2)	8186 (35.4)	<.001
Septicemia	14 560 (16.8)	3139 (16.2)	3568 (16.3)	3690 (16.8)	4163 (18.0)	<.001
Major trauma	4332 (5.0)	743 (3.8)	1159 (5.3)	1018 (4.6)	1412 (6.1)	<.001
Metabolic or electrolyte abnormality	21 673 (25.1)	3692 (19.0)	4912 (22.5)	5263 (23.9)	7806 (33.7)	<.001
Metastatic or hematologic cancer	9220 (10.7)	1733 (8.9)	2226 (10.2)	2449 (11.1)	2812 (12.1)	<.001
Interventions in place						
Mechanical ventilation	36 134 (41.8)	8087 (41.6)	9160 (41.9)	8989 (40.9)	9898 (42.7)	<.001
Vasoactive agent	22 620 (26.2)	4451 (22.9)	5743 (26.3)	5716 (26.0)	6710 (29.0)	<.001
Dialysis	2608 (3.0)	434 (2.2)	519 (2.4)	511 (2.3)	1144 (4.9)	<.001
Intraarterial catheter	9400 (10.9)	1267 (6.5)	1845 (8.4)	2384 (10.8)	3904 (16.9)	<.001

Abbreviations: CNS, central nervous system; IQR, interquartile range.

^a^ Data are presented as number (percentage) of patients unless otherwise indicated.

^b^ Continuous variables were compared using 1-way analysis of variance. Categorical variables were compared using χ^2^ or Fisher exact test.

**Table 2.** Hospital Characteristics^a^

Characteristic	No./Total No. (N = 290)	Overall risk-standardized survival rate, No./Total No.				*P* value^b^
		Quartile 1 (n = 72)	Quartile 2 (n = 73)	Quartile 3 (n = 73)	Quartile 4 (n = 72)	
Beds, No.						.08
<200	46/230 (20.0)	11/59 (18.6)	7/55 (12.7)	17/55 (30.9)	11/61 (18.0)	
200-499	122/230 (53.0)	37/59 (62.7)	31/55 (56.4)	26/55 (47.3)	28/61 (45.9)	
≥500	62/230 (27.0)	11/59 (18.6)	17/55 (30.9)	12/55 (21.8)	22/61 (36.1)	
Missing	60	13	18	18	11	
Cardiac beds, No.						.01
0	65/214 (30.4)	16/56 (28.6)	13/52 (25.0)	25/52 (48.1)	11/54 (20.4)	
1-10	41/214 (19.2)	9/56 (16.1)	13/52 (25.0)	7/52 (13.5)	12/54 (22.2)	
11-20	50/214 (23.4)	21/56 (37.5)	11/52 (21.2)	8/52 (15.4)	10/54 (18.5)	
21-30	31/214 (14.5)	7/56 (12.5)	10/52 (19.2)	5/52 (9.6)	9/54 (16.7)	
≥31	27/214 (12.6)	3/56 (5.4)	5/52 (9.6)	7/52 (13.5)	12/54 (22.2)	
Missing	76	16	21	21	18	
Intensive care beds, No.						.67
0	8/214 (3.7)	1/56 (1.8)	2/52 (3.8)	3/52 (5.8)	2/54 (3.7)	
≤12	35/214 (16.4)	11/56 (19.6)	7/52 (13.5)	12/52 (23.1)	5/54 (9.3)	
13-25	80/214 (37.4)	24/56 (42.9)	21/52 (40.4)	16/52 (30.8)	19/54 (35.2)	
26-50	65/214 (30.4)	16/56 (28.6)	16/52 (30.8)	13/52 (25.0)	20/54 (37.0)	
>50	26/214 (12.1)	4/56 (7.1)	6/52 (11.5)	8/52 (15.4)	8/54 (14.8)	
Missing	76	16	21	21	18	
Academic status						.15
Major teaching	68/233 (29.2)	14/59 (23.7)	18/56 (32.1)	11/56 (19.6)	25 (40.3)	
Minor teaching	73/233 (31.3)	21/59 (35.6)	20/56 (35.7)	17/56 (30.4)	15 (24.2)	
Nonteaching	92/233 (39.5)	24/59 (40.7)	18/56 (32.1)	28/56 (50.0)	22 (35.5)	
Missing	57	13	17	17	10	
Location						.98
Rural	16/233 (6.9)	4/59 (6.8)	3/56 (5.4)	4/56 (7.1)	5 (8.1)	
Urban	217/233 (93.1)	55/59 (93.2)	53/56 (94.6)	52/56 (92.9)	57 (91.9)	
Missing	57	13	17	17	10	
US Census region						.02
North Mid-Atlantic	42/234 (17.9)	14/59 (23.7)	8/57 (14.0)	7/56 (12.5)	13/62 (21.0)	
South Atlantic	60/234 (25.6)	14/59 (23.7)	15/57 (26.3)	16/56 (28.6)	15/62 (24.2)	
North Central	53/234 (22.6)	5/59 (8.5)	12/57 (21.1)	13/56 (23.2)	23/62 (37.1)	
South Central	36/234 (15.4)	14/59 (23.7)	8/57 (14.0)	11/56 (19.6)	3/62 (4.8)	
Mountain Pacific	43/234 (18.4)	12/59 (20.3)	14/57 (24.6)	9/56 (16.1)	8/62 (12.9)	
Missing	56	13	16	17	10	
Cardiopulmonary arrest events, No.						.67
Mean (SD)	298.0 (252.3)	269.8 (208.6)	299.4 (222.9)	301.2 (287.9)	321.6 (282.4)	
Median (IQR)	234.0 (109.0-393.0)	222.5 (124.0-376.0)	256.0 (111.0-459.0)	203.0 (90.0-375.0)	249.5 (125.0-401.0)	

Abbreviation: IQR, interquartile range.

^a^ Data are presented as number (percentage) of patients unless otherwise indicated.

^b^ Continuous variables were compared using 1-way analysis of variance. Categorical variables were compared using χ^2^ or Fisher exact test.

Among study hospitals, the median RSSR was 25.1% (IQR, 21.9%-27.7%; range, 14.1%-40.8%), with substantial variation across sites (eFigure 2 in the Supplement). The median odds ratio for RSSR was 1.36 (95% CI, 1.31-1.40), suggesting that the odds of survival for a patient with IHCA would be 36% higher at 1 randomly selected hospital compared with another randomly selected hospital after adjustment for differences in case mix across sites. Given this variability, we categorized study hospitals into quartiles based on the risk-standardized survival metric: Q1 (<21.9%), Q2 (21.9%-25.2%), Q3 (25.3%-27.7%), and Q4 (>27.7%).

Patient characteristics across RSSR quartiles are also shown in Table 1, with hospital characteristics shown in Table 2. Patients in Q1 hospitals were more likely to be black (31.2% vs 18.7%) and to have an initial nonshockable (asystole or pulseless electrical activity) rhythm (85.8% vs 81.5%) compared with patients in Q4 hospitals. Patients in Q1 hospitals were less likely to be receiving intravenous vasopressors (22.9% vs 29.0%) or dialysis before the cardiac arrest (2.2% vs 4.9%) (*P* < .001 for all). In general, the prevalence of most comorbidities was higher among patients at Q4 hospitals compared with patients at Q1 hospitals. For hospital characteristics, the proportion of cardiac beds and census region were the only variables associated with hospital survival quartile. Q4 hospitals had more cardiac beds and were more likely to be located in the North Central region compared with Q1 hospitals.

Table 3 shows rates of acute resuscitation survival and postresuscitation survival for all hospitals and across hospital quartiles. The median risk-adjusted rate of acute resuscitation survival (ie, patients who achieved return of spontaneous circulation) was 72.4% (IQR, 67.9%-76.9%; range, 46.0%-84.7%; median odds ratio, 1.40; 95% CI, 1.35-1.45) (eFigure 3 in the Supplement). Patients at Q4 hospitals had a mean acute resuscitation survival rate of 75.4%, compared with a mean acute resuscitation survival rate of 66.8% for patients at Q1 hospitals (absolute difference, 8.5%; 95% CI, 6.6%-10.5%; *P* < .001). Among Q1 hospitals, 50.0% (36 of 72) were in the corresponding quartile of risk-adjusted acute resuscitation survival, and among Q4 hospitals, 45.8% (33 of 72) were in the corresponding quartile (Table 4).

**Table 3.** Risk-Standardized Survival Rates of Acute Resuscitation and Postresuscitation Survival Across Hospital Quartiles

Survival Type	Overall risk-standardized survival rate			
	Quartile 1 (n = 72)	Quartile 2 (n = 73)	Quartile 3 (n = 73)	Quartile 4 (n = 72)
Acute resuscitation, %				
Mean (SD)	66.8 (6.4)	72.5 (5.1)	73.6 (5.5)	75.4 (5.5)
Median (IQR)	67.9 (62.9-71.0)	72.6 (68.9-76.6)	74.3 (70.2-77.5)	76.4 (72.2-79.4)
Postresuscitation, %				
Mean (SD)	28.7 (3.4)	32.6 (2.0)	35.8 (2.5)	40.3 (3.2)
Median (IQR)	28.8 (26.2-31.1)	32.6 (31.5-33.8)	35.4 (34.0-37.3)	40.0 (38.2-41.5)

Abbreviation: IQR, interquartile range.

**Table 4.** Association Between Hospital’s Performance on the Risk-Standardized Survival Rates With Acute Resuscitation Survival and Postresuscitation Survival

Survival type	Overall risk-standardized survival rate, No. (%)			
	Quartile 1	Quartile 2	Quartile 3	Quartile 4
Acute resuscitation survival quartile				
1 (n = 72)	36 (50.0)	15 (20.5)	12 (16.7)	9 (12.5)
2 (n = 73)	23 (31.9)	20 (27.4)	20 (27.4)	10 (13.7)
3 (n = 73)	11 (15.3)	23 (31.5)	19 (26.0)	20 (27.4)
4 (n = 72)	2 (2.8)	15 (20.8)	22 (30.6)	33 (45.8)
Postresuscitation survival quartile				
1 (n = 72)	55 (76.4)	17 (23.6)	0	0
2 (n = 73)	12 (16.4)	41 (56.2)	19 (26.0)	1 (1.4)
3 (n = 73)	5 (6.9)	14 (19.2)	39 (53.4)	15 (20.6)
4 (n = 72)	0	1 (1.4)	15 (20.8)	56 (77.8)

The median risk-adjusted rate of postresuscitation survival (ie, survival to discharge among patients with return of spontaneous circulation) was 34.0% (IQR, 31.5%-37.7%; range, 21.4%-50.4%; median odds ratio, 1.35; 95% CI, 1.30-1.40) (eFigure 4 in the Supplement). Patients at Q4 hospitals had a mean risk-adjusted postresuscitation survival rate of 40.3%, compared with 28.7% for patients at Q1 hospitals (absolute difference, 11.5%; 95% CI, 10.5%-12.7%; *P* < .001). Of Q1 hospitals, 76.4% (55 of 72) were also categorized in Q1 of risk-adjusted postresuscitation survival; similarly, of Q4 hospitals, 56 of 72 (76.8%) were categorized in Q4 of risk-adjusted postresuscitation survival (Table 4).

The Figure shows the correlation between hospital RSSR, acute resuscitation survival, and postresuscitation survival. Although hospital rates of RSSR were correlated with both survival during both phases, the correlation between a hospital’s overall RSSR and postresuscitation survival was stronger (ρ, 0.90; *P* < .001) (Figure, A) compared with the correlation with acute resuscitation survival (ρ, 0.50; *P* < .001) (Figure, B). There was no correlation between hospital risk-adjusted rates of acute resuscitation survival and post-resuscitation survival (ρ, 0.09; *P* = .11) (Figure, C).

**Figure.**
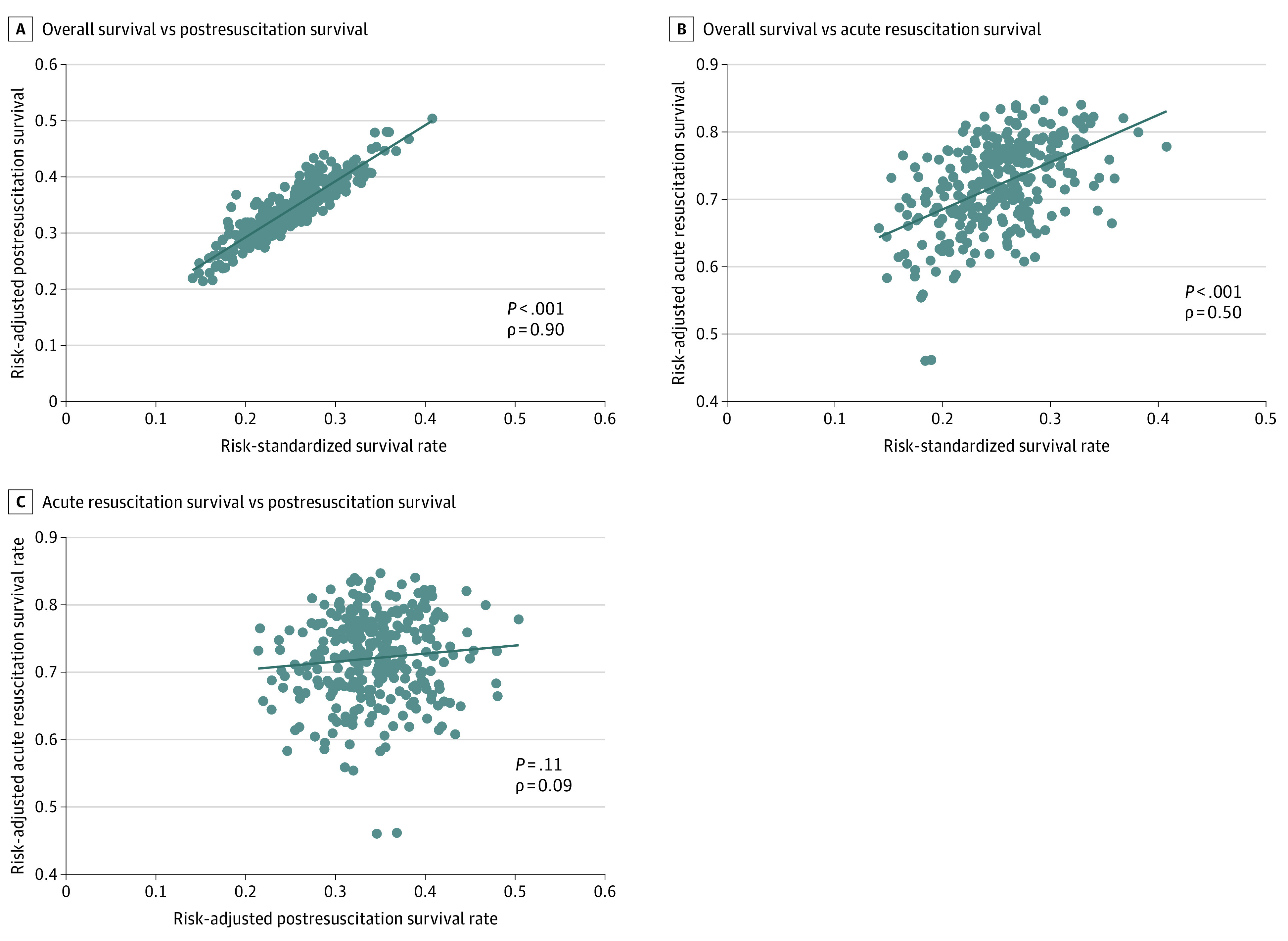
Correlation Between Overall Risk-Standardized Survival Rate, Acute Resuscitation Survival, and Postresuscitation Survival

## Discussion

In this contemporary study of 290 GWTG-Resuscitation hospitals, we found an approximately 3-fold variation in overall rates of IHCA survival (14.1%-40.8%). Although we found that a hospital’s rate of overall survival was correlated with both acute resuscitation and postresuscitation survival, the correlation with postresuscitation survival was stronger (ρ, 0.90 vs 0.50). In addition, we found no correlation between a hospital’s rate of acute resuscitation and postresuscitation survival. These findings suggest that hospitals with the highest IHCA survival rates, in general, excelled in either acute resuscitation survival or postresuscitation survival but did not consistently excel in both phases of care. Collectively, our findings have important implications for the design of hospital-based quality improvement initiatives that largely focus on acute resuscitation care.

The strength of correlation between overall IHCA survival and postresuscitation survival has important implications for ongoing quality improvement efforts. The current GWTG-Resuscitation award system that recognizes hospitals for high quality resuscitation is entirely composed of metrics based on acute resuscitation care and includes (1) time from cardiac arrest to initiation of chest compressions, (2) time from cardiac arrest to first defibrillation, (3) device confirmation of endotracheal tube placement, and (4) whether a cardiac arrest was monitored or witnessed by hospital personnel. Use of these metrics may explain why a previous study found no association between hospitals’ performance and their risk-standardized survival.^16^ Thus, an incentive strategy focused on acute resuscitation care alone would be limited in reducing hospital variation in IHCA survival or increasing overall survival. Our study highlights the need to develop and validate hospital strategies that distinguish top-performing hospitals in postresuscitation care.

The development of quality metrics for postresuscitation care has been substantially hampered by the lack of evidence from randomized clinical trials for existing postarrest treatments. For example, clinical trials have largely shown a benefit of targeted temperature management (TTM) only in patients with out-of-hospital cardiac arrest.^17,18,19^ Observational studies of TTM in adults with IHCA have also yielded mixed results, with the largest one showing no survival benefit.^20,21,22^ A dedicated randomized clinical trial of TTM in patients with IHCA was conducted in children and did not show a benefit.^23^ However, a recent randomized clinical trial of patients with cardiac arrest due to a nonshockable rhythm that included 27% of patients with IHCA found higher rates of favorable neurologic survival in patients treated with moderate therapeutic hypothermia compared with targeted normothermia.^24^ Likewise, a strategy of routine coronary angiography, to date, has not been shown to be associated with improved survival in patients successfully resuscitated from out-of-hospital cardiac arrest,^25^ but remains to be studied in patients with IHCA.

Although TTM and routine coronary angiography remain therapeutic options that require further study, hospitals that excel in postresuscitation care are more likely to structure and deliver high-quality care to successfully resuscitated patients in the ICU. The American Heart Association recommends a multipronged strategy focused on optimization of hemodynamics, gas exchange, neurologic and metabolic parameters with care guided by specialists in intensive care, neurocritical care, and cardiology.^26^ Although best practices for maximizing postresuscitation survival have not been clearly delineated, a few medical centers have developed highly specialized postcardiac arrest care teams that provide consultation 24 hours per day for 7 days per week to all patients with cardiac arrest throughout the hospital.^27^ Such a team-based structure ensures that management of these patients needing complex care is concentrated among a small group of physicians with appropriate expertise and that care is standardized according to protocols. It is important to determine how the use of innovative postarrest strategies such as the use of specialized cardiac arrest teams is associated with postresuscitation and overall IHCA survival. Because existing registries such as GWTG-Resuscitation do not capture these data, identifying best practices for postresuscitation survival will require a combination of quantitative and qualitative approaches (ie, mixed methods) to identify best practices for improving postresuscitation and overall IHCA survival.

### Limitations

This study has limitations. First, hospitals participating in GWTG-Resuscitation are predominantly large, urban hospitals with an interest in resuscitation quality improvement, which may limit the generalizability of our findings. Second, although GWTG-Resuscitation collects rich data on patient-level variables for case-mix adjustment, the potential for residual confounding because of unmeasured clinical or socioeconomic variables remains. Third, we lacked information on postresuscitation treatment strategies at individual hospitals, which limited our ability to identify the specific hospital practices that may be associated with hospital performance on postresuscitation. Fourth, our study was primarily limited to in-hospital survival outcomes and data on quality of life; data on physical and mental functioning after hospital discharge were not available.

## Conclusions

The findings suggest that hospitals with high overall survival rates for IHCA, in general, excel in either acute resuscitation or postresuscitation care but not both. Since most hospital-based quality improvement initiatives largely focus on acute resuscitation survival, our findings suggest that efforts to strengthen postresuscitation intensive care may offer additional opportunities to improve IHCA survival.

## Notes

Supplement. **eFigure 1.** Study Cohort **eFigure 2.** Hospital Variation in Overall Risk-Standardized Survival (RSSR) to Discharge **eFigure 3.** Hospital Variation in Risk-Adjusted Rate of Acute Resuscitation Survival **eFigure 4.** Hospital Variation in Risk-Adjusted Rate of Postresuscitation Survival
