# Supplementary material for: Association of Hospital-Level Acute Resuscitation and Postresuscitation Survival With Overall Risk-Standardized Survival to Discharge for In-Hospital Cardiac Arrest
Source: JAMA Netw Open. 2020 Jul 10;3(7):e2010403. doi: 10.1001/jamanetworkopen.2020.10403 (PMC7352153; doi:10.1001/jamanetworkopen.2020.10403)
Supplement: Supplement. — eFigure 1. Study Cohort eFigure 2. Hospital Variation in Overall Risk-Standardized Survival (RSSR) to Discharge eFigure 3. Hospital Variation in Risk-Adjusted Rate of Acute Resuscitation Survival eFigure 4. Hospital Variation in Risk-Adjusted Rate of Postresuscitation Survival [file jamanetwopen-3-e2010403-s001.pdf]

Supplementary Online Content

Girotra S, Nallamothu BK, Tang Y, Chan PS, et al; American Heart Association Get With The Guidelines–Resuscitation Investigators. Association of hospital-level acute resuscitation and postresuscitation survival with overall risk-standardized survival to discharge for in-hospital cardiac arrest. *JAMA Netw Open*. 2020;3(7):e2010403. doi:10.1001/jamanetworkopen.2020.10403

eFigure 1. Study Cohort

eFigure 2. Hospital Variation in Overall Risk-Standardized Survival (RSSR) to Discharge

eFigure 3. Hospital Variation in Risk-Adjusted Rate of Acute Resuscitation Survival

eFigure 4. Hospital Variation in Risk-Adjusted Rate of Postresuscitation Survival

This supplementary material has been provided by the authors to give readers additional information about their work.

© 2020 Girotra S, et al. *JAMA Network Open.*

eFigure 1. Study Cohort

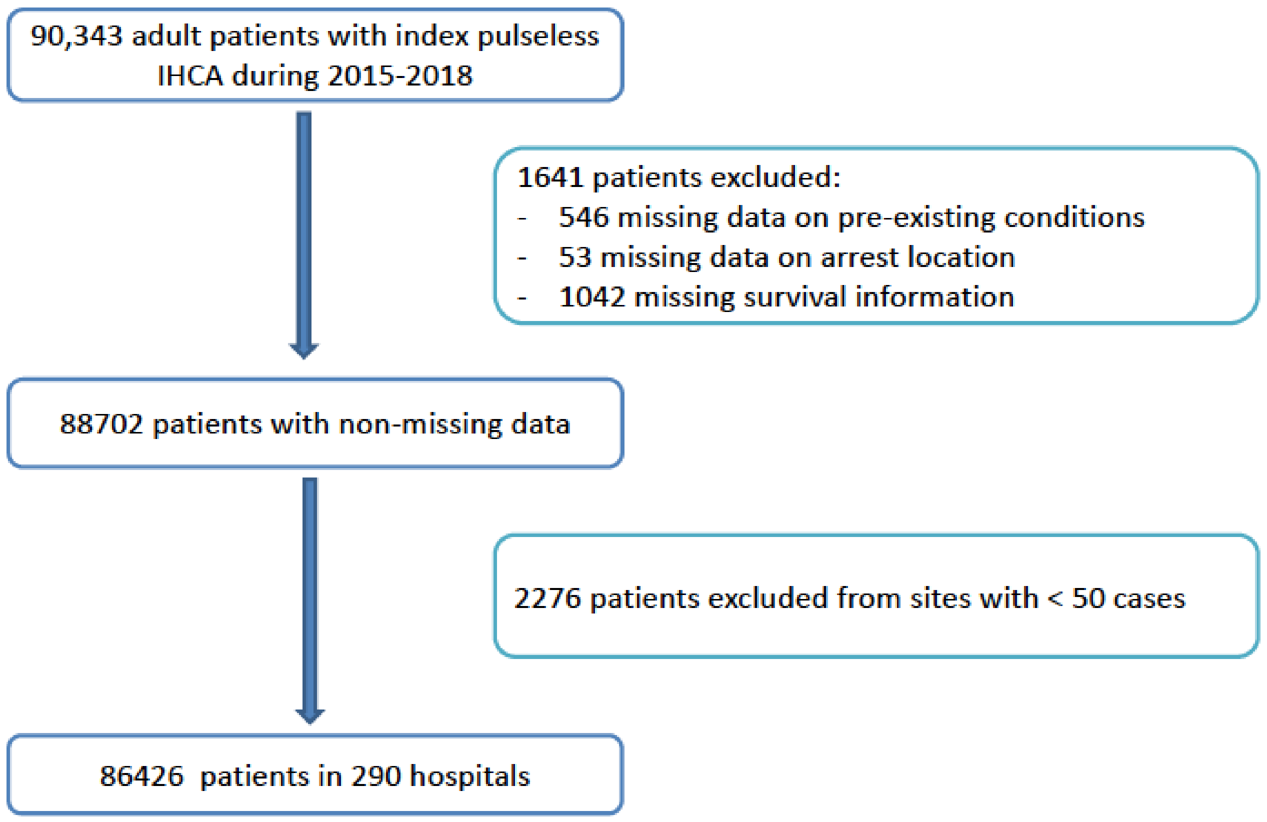

© 2020 Girotra S, et al. *JAMA Network Open.*

eFigure 2. Hospital Variation in Overall Risk-Standardized Survival (RSSR) to Discharge

The median risk-standardized survival to discharge was 25.1% (inter-quartile range: 21.9%-27.7%; range:14.1%-40.8% median odds ratio 1.36; 95% CI: 1.31-1.40).

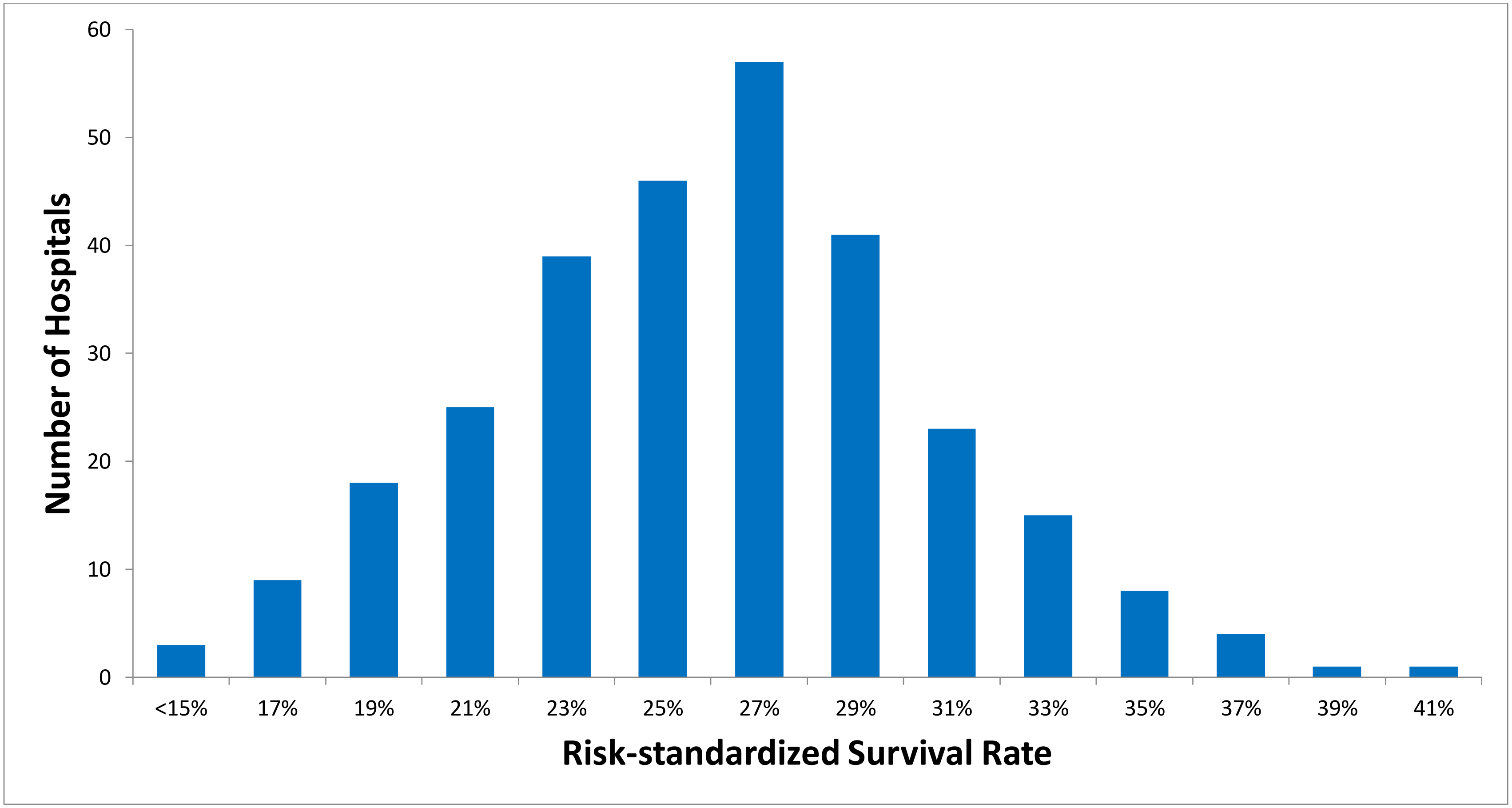

© 2020 Girotra S, et al. *JAMA Network Open.*

eFigure 3. Hospital Variation in Risk-Adjusted Rate of Acute Resuscitation Survival

The median risk-adjusted rate of acute resuscitation survival was 72.4% (inter-quartile range: 67.9%-76.9%; range: 46.0%-84.7%; median OR 1.40; 95% CI 1.35-1.45)

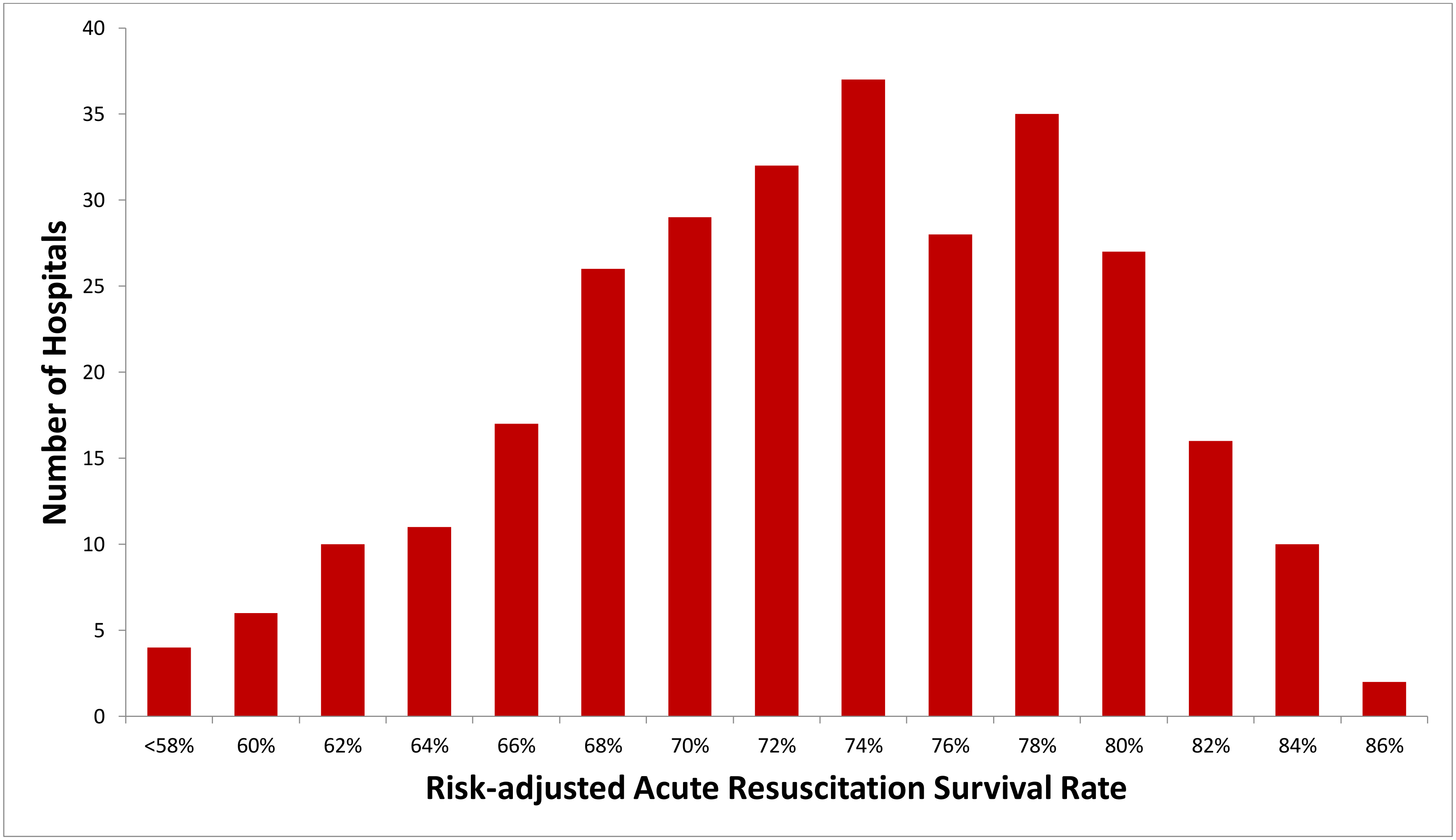

© 2020 Girotra S, et al. *JAMA Network Open.*

eFigure 4. Hospital Variation in Risk-Adjusted Rate of Postresuscitation Survival

The median risk-adjusted rate of post resuscitation survival was 34.0% (inter-quartile range: 31.5%-37.7%; range: 21.4%-50.4%; median OR 1.35 95% CI 1.30-1.40)

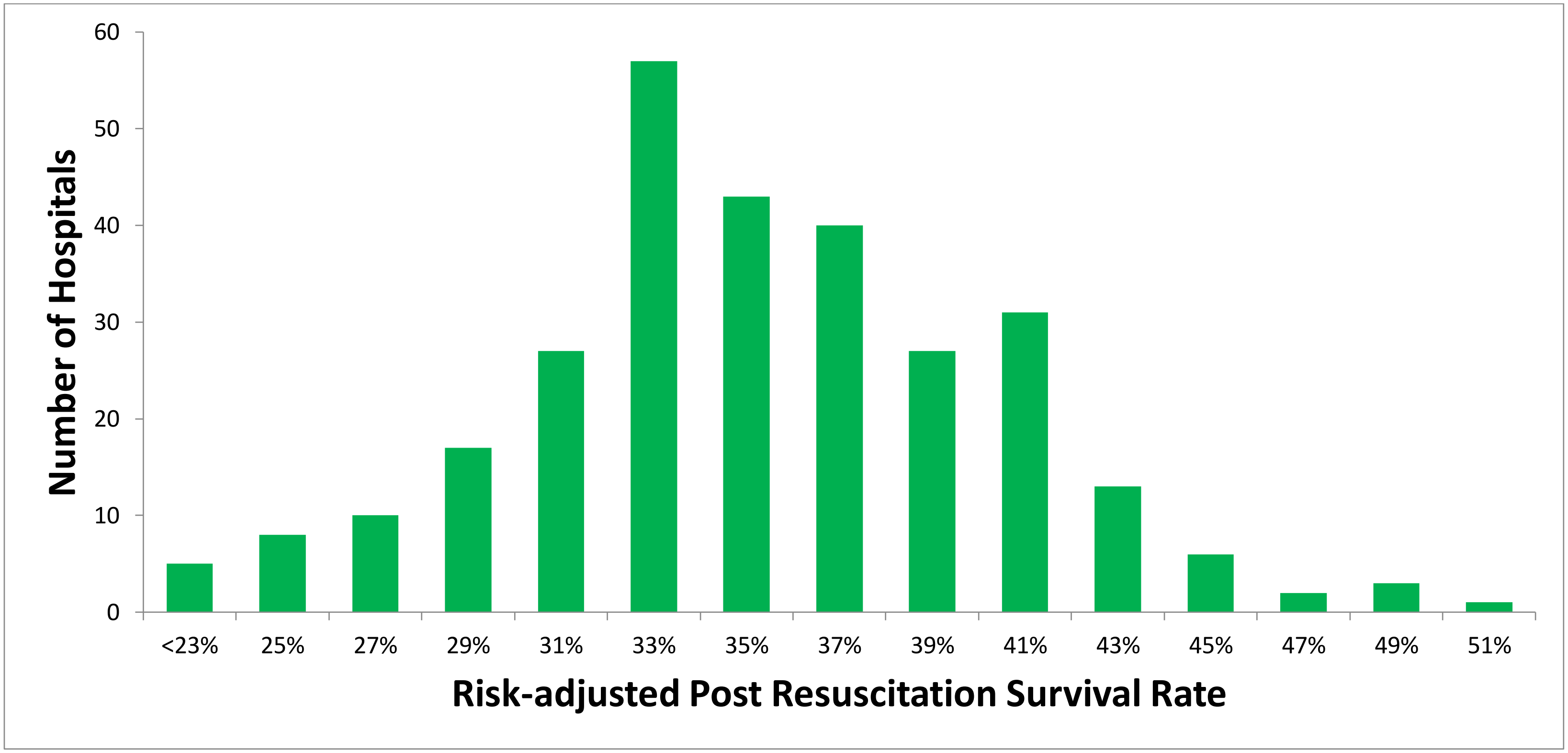

© 2020 Girotra S, et al. *JAMA Network Open.*
